# Epitopes of Immunoreactive Proteins of *Streptococcus Agalactiae*: Enolase, Inosine 5′-Monophosphate Dehydrogenase and Molecular Chaperone GroEL

**DOI:** 10.3389/fcimb.2018.00349

**Published:** 2018-10-02

**Authors:** Anna Dobrut, Ewa Brzozowska, Sabina Górska, Marcelina Pyclik, Andrzej Gamian, Małgorzata Bulanda, Elzbieta Majewska, Monika Brzychczy-Włoch

**Affiliations:** ^1^Department of Molecular Medical Microbiology, Chair of Microbiology, Faculty of Medicine, Jagiellonian University Medical College, Krakow, Poland; ^2^Ludwik Hirszfeld Institute of Immunology and Experimental Therapy, Polish Academy of Sciences, Wroclaw, Poland; ^3^Department of Clinical Obstetrics and Perinatology, University Hospital, Krakow, Poland

**Keywords:** *Streptococcus agalactiae*, immunogenic proteins, epitope mapping, enolase, inosine 5′-monophosphate dehydrogenase IMPDH, molecular chaperone GroEL, diagnostic test

## Abstract

Three *Streptococcus agalactiae* (group B streptococci, GBS) immunoreactive proteins: enolase (47.4 kDa), inosine 5′-monophosphate dehydrogenase (IMPDH) (53 kDa) and molecular chaperone GroEL (57 kDa) were subjected to investigation. Enolase protein was described in our previous paper, whereas IMPDH and GroEL were presented for the first time. The aim of our paper was to provide mapping of specific epitopes, highly reactive with umbilical cord blood serum. Bioinformatic analyses allowed to select 32 most likely epitopes for enolase, 36 peptides for IMPDH and 41 immunoreactive peptides for molecular chaperone GroEL, which were synthesized by PEPSCAN. Ten peptides: two in enolase, one in IMPDH and seven in molecular chaperone GroEL have been identified as potentially highly selective epitopes that can be used as markers in rapid immunological diagnostic tests or constitute a component of an innovative vaccine against GBS infections.

## Introduction

*Streptococcus agalactiae* (Group B Streptococcus, GBS) is a Gram-positive opportunistic pathogen, which colonizes the genitourinary and gastrointestinal tracts. However, infections caused by this bacterium can be dangerous for some patients, including newborns, pregnant women, elderly patients, diabetics, and immunosuppressed adults (Edwards and Baker, 2005; Rodriguez-Granger et al., 2012). The percentage of carriers among pregnant European women varies and ranges from 6.6% in Greece to 36% in Denmark (Tsolia et al., 2003; Hansen et al., 2004). In Poland, depending on the methodology, up to 30% of pregnant women are estimated to be colonized by GBS (Brzychczy-Włoch et al., 2012). The risk of transmission to infants during labor reaches 70% and the probability of infection onset are 2–4 per 1,000 live births (Wessels and Kasper, 1998). Infections caused by GBS typically take the form of sepsis, pneumonia, or meningitis, which result in high mortality among infants (Johri et al., 2006). To decrease the growing number of GBS carriage, since 1996, according to the Centers for Disease Control and Prevention, pregnant women at 35–37 weeks' gestation are obliged to be screened for GBS carriage. The detection procedure is based on screening of swabs taken from the vagina and the rectum, followed by cultivation on blood agar medium and, if applicable, antibiotic therapy is introduced. Women who have at least one risk factor from the CDC's definition are also prescribed medication (Verani et al., 2010). An alternative detection method for rapid diagnosis in pregnant women is based on the PCR protocol. However, its widespread use is limited because of reagent costs and apparatus requirements (Verani et al., 2010).

There are numerous immunoreactive GBS proteins recognized by protective antibodies, which could be markers of infections caused by *S. agalactiae* as well as components of innovative subunit vaccine (Baker and Edwards, 2003). The best-known immunoreactive GBS proteins are the conservative and chimeric ones belonging to the alpha-like protein (Alp) family (Lindahl et al., 2003). Other recently investigated proteins, including the fibrinogen-binding protein FsbA, peptidase C5a, the laminin-binding protein Lmb, the conservative protein Sip and immunogenic bacterial adhesin BibA, demonstrate their immunoreactive nature and are considered potential vaccine components (Johri et al., 2006; Rajagopal, 2009; Santillan et al., 2011; Dzanibe et al., 2016). It was shown that BibA ensures 69% protection against a lethal challenge dose of GBS serotype-III in mice models (Santi et al., 2009). Trials on vaccine candidates, such as the Rib protein, which belongs to the Alp family, or pilus proteins have been in the preclinical phase (Heath, 2016).

In our previous research, four immunogenic proteins, including: enolase (47.4 kDa), aldehyde dehydrogenase (50.6 kDa) and, firstly described as being immunogenic in GBS, the trigger factor (47 kDa) and elongation factor Tu (44 kDa) have been described (Brzychczy-Wloch et al., 2013). In the present paper, we identified two more immunogenic proteins: inosine 5′-monophosphate dehydrogenase (IMPDH) (53 kDa) and the molecular chaperone GroEL (57 kDa).

The aim of this paper was to provide mapping of the epitopes of three immunoreactive GBS proteins: enolase, IMPDH and molecular chaperone GroEL using PEPSCAN method. The epitopes were chemically synthesized on polyethylene pins and their immunospecificity was tested in the presence of both GBS-positive and GBS-negative sera. The identified epitopes might be used in the future as an immunoassay for diagnostic infections caused by GBS as well as a component of a vaccine against GBS infections.

## Materials and methods

### Specimen collection

The study was approved by Jagiellonian University Bioethical Committee decision No. KBET/153/B/2014. The study included 34 pregnant women that were patients of the Department of Clinical Obstetrics and Perinatology, University Hospital in Krakow, Poland. The consent obtained from the participants was both informed and written. The inclusion criteria: pregnant women in the third trimester between 18 and 40 years of age, a written statement of consent to participate in the study. The exclusion criteria: pregnant women below 18 and over 40 years of age, patients with immunodeficiency or autoimmune diseases, pregnant women with the so-called high-risk pregnancy or with perinatal complications, preterm delivery, no written consent to participate in the study or its withdrawal (Pyclik et al., 2018). The patients included in the study had been divided into two groups: I. Study group—women with confirmed GBS colonization (n = 20); II. Control group—women not colonized by GBS (*n* = 14).

All the studied patients were tested for the presence of *Streptococcus agalactiae* by swabs taken from the lower vagina (vaginal introitus) and the rectum (anal sphincter) between the 35th and 37th week of gestation. The samples collected were cultured and identified in accordance with the Centers for Disease Control and Prevention guidelines (Verani et al., 2010) [8].

Umbilical cord blood samples (50 ml) were collected after successful labor. Serum samples isolated from the blood collected were stored at −70°C. To qualify serum as GBS-positive or GBS-negative, the immunoblot with the presence of GBS immunogenic proteins was done. High immunoreactivity observed which was measured by absorbance value in ELISA assay, qualified serum as GBS-positive, whereas low immunoreactivity qualified serum as GBS-negative. Additionally, we included venous blood serum from adult patients to type the most immunoreactive GBS proteins.

### Bacterial strains

*S. agalactiae* isolates (*n* = 180) came from the study group of pregnant women (*n* = 20), from the patients with urinary tract infections (UTI) (*n* = 100) and strains from the pregnant, non-pregnant patients, and newborns, with different clinical status (infection and carrier), isolated from various material, such as blood, vagina and anus swabs, newborn ear swab and, urine (*n* = 60) collected within the framework of two projects financed by the Polish Ministry of Research and Higher Education and no. 3PO5E08425 and NN401042337 and described in details in our previous paper (Brzychczy-Wloch et al., 2013). The isolates tested represented various serotypes (Ia, Ib, II-V), different genes coding surface of the Alpha-like proteins (*bca, alp2, alp3, epsilon, rib*), different macrolide resistance phenotypes (cMLSB, iMLSB, M), various sequence types (ST) and came from different regions of Poland. Identification of the bacterial species was performed by PCR with species-specific primers (Ke et al., 2000). Molecular characterization of the tested GBS strains was based on the methodology described in our previous work (Brzychczy-Wloch et al., 2013). All strains were stored at −70°C.

### Protein preparation and analysis

Bacterial isolates were grown on a brain-heart infusion broth (BHI, Biocorp) for 24 h at 37°C in aerobic conditions. Afterwards, cultures in final concentration in solution A600 nm = 1.0 were subjected to further processing, which included suspension bacterial pellet in Tris-HCl (Merck) containing various sodium dodecyl sulfate concentration (1–2%) (Sigma-Aldrich) or directly in the electrophoresis buffer according to Heilmann, however, boiling was skipped (Heilmann et al., 1996). Next, samples were sonicated three times for 5 min, and centrifuged for 1 min in PBS. Proteins were precipitated from the supernatant obtained using 3 volumes of cold 95% ethanol (POCH) and incubated at 4°C overnight. In the next step, the precipitated proteins were centrifuged, dissolved in water and their concentration was measured according to the BCA assay (Smith et al., 1985).

### Immunoblotting

The proteins were separated in SDS-PAGE using Prep-Cell apparatus (Model 491 Bio-Rad) and transferred to the Immobilon-P membrane (MILIPORE) and subjected to immunoblotting. The membranes were blocked in PBS containing 1% of bovine serum albumin (BSA, Thermo Fisher Scientific) for 1 h, and washed three times with PBS-T [PBS containing 0.25% Tween (Sigma-Aldrich)]. Next, the membranes were incubated with human sera, both GBS-positive and GBS-negative, in 1:300 dilution for 2 h at 37°C and washed three times with PBS-T. Afterwards, membranes were incubated with alkaline phosphatase-conjugated goat anti-human IgG antibodies (Sigma-Aldrich) diluted 1:5000 for 1 h in the room temperature. Eventually, after the triple washing with PBST, membranes were submerged in solution containing nitroblue tetrazolium (NBT, Roth), 5-bromo-4-chloro-3-indolyl phosphate (BCIP, Roth), and MgCl_2_ (POCh) was added for 5 s to visualize reaction. Bands of interest were cut out from gel and subjected to digestion by using a proteolytic enzyme such as trypsin (Roche) to obtain a mixture of peptides. Then, the peptides were separated by liquid chromatography (LC), and mass fragments were measured using mass spectrometer LC-MS/MS Orbitrap (Thermo). Eventually, proteins were identified by comparative analysis of peptides masses (NCBI, UniProt databases) using MASCOT (http://www.matrixscience.com/) and statistical analysis.

### Bioinformatics

Among 180 tested GBS isolates, we found that six proteins, which molecular masses were: 44, 47, 47.4, 50.6, 53, 57 kDa, which, in immunoblot, showed in every examined strain reacted with GBS-positive serum, whereas no reactivity was noticed in presence of GBS-negative serum. Next, bands from three representative, randomly selected GBS strains were subjected to bioinformatic analyses were subjected to bioinformatic analyses: (1) *S. agalactiae* D129 (serotype III, *rib* gene, sequence type ST170) isolated from a patient with urinary tract infection (UTI); (2) *S. agalactiae* 1736/08 (serotype V, *alp2* gene, cMLSB phenotype, *erm*B gene, sequence type ST1), isolated from a newborn with UTI; (3) *S. agalactiae* 13793/08 (serotype V, *alp3* gene, cMLSB phenotype, *erm*B gene, sequence type ST1) isolated from a newborn with UTI. Each protein demonstrated high immunoreactivity to GBS-positive sera, however, they barely reacted with GBS-negative sera. Epitope prediction was based on amino acid sequence analysis. Prediction of the protein's secondary structure, loop regions, burial, and disorder of amino acids was carried out using Genesilico Metaserver (https://genesilico.pl/meta2). Antibody Epitope Prediction web server (http://tools.immuneepitope.org/) was used for epitope localization within the tested protein. Other methods applied to predict the most probable epitopes among the investigated immunoreactive proteins were: Emini Surface Accessibility Prediction (Emini et al., 1985), Kolaskar & Tongaonkar Antigenicity (Kolaskar and Tongaonkar, 1990), Bepipred Linear Epitope Prediction (Larsen et al., 2006). B-cell epitope prediction was done using another cross method, BCPREDS (http://ailab.ist.psu.edu/). Protein modeling was based on the crystal structure: for enolase−1W6T (crystal structure of octameric enolase from *Streptococcus pneumoniae*), for IMPDH−1ZFJ (inosine monophoshate dehydrogenase (IMPDH; EC 1.1.1.205) from *Streptococcus pyogenes*), and for molecular chaperone GroEL - 3RTK (Crystal structure of Cpn60.2 from *Mycobacterium tuberculosis* at 2.8A). For more detailed prediction, homology models made based on 3D structure were done. That allowed the identification of loop regions on the surface proteins with disorder tendency which can indicate the most probable epitope localization. Sequences which had been identified as probable epitopes by at least two prediction methods and consisted of at least 6 amino acids were chosen for further analyses.

### Peptide synthesis

Peptide synthesis was carried out according to a slightly modified Geysen's procedure (PEPSCAN) (Geysen et al., 1987; Pyclik et al., 2018) on the NCP Block of 96 hydroxypropylmethacrylate pins (MIMOTOPES), following the one-plate-one-peptide approach. Stepwise elongation of peptides from C-end to N-end was carried out for 6 h or at night in the presence of coupling solution which contained 60 mM Fmoc corresponding to amino acids diluted in N,N'-dimethylformamide (DMF, Merck KGaA), two coupling reagents: 65 mM of 1-hydroxy-7-azabenzotriazole (Sigma-Aldrich) and 60 mM of diisopropylcarbodiimide (Merck KGaA) and 10 mM bromophenol blue (Sigma-Aldrich). After completion of the synthesis, deprotection was performed. Eventually, pins were subjected to disruption in a buffer containing 1% SDS (Sigma-Aldrich), 0.1% 2-mercaptoethanol (Thermo Fisher) and 0.1 M Na3PO4 (Sigma-Aldrich) of pH = 7.2, heated to 60°C in a sonicator (Branson 2210 DTH Ultrasonic Cleaner) and sonicated for 10 min. The disruption buffer was removed from the pins by submersion in MiliQ water warmed to 60°C for 2 min, followed by washing in MeOH (CHEMPUR) warmed to 60°C for 5 min. Afterwards, the pins were fully dried and kept in dry conditions at 4°C or −20°C.

### ELISA assay

The ELISA assay was performed according to the procedure described in our previous paper (Pyclik et al., 2018). The procedure was repeated at least three times for every amino acid sequence, both with GBS-positive and GBS-negative sera. Epitope detection was carried out in the presence of pooled both GBS-positive and GBS-negative sera, which resulted from the limitation of the PEPSCAN method, such as the use of polyethylene pins in consecutive ELISA assays. Nevertheless, the specificity of the epitopes identified was confirmed with the presence of individual sera: 10 for GBS-positive (1/KP, 2/KP, 3/KP, 4/KP, 5/KP, 6/KP, 8/KP, 10/KP, 14/KP, 15/KP) and 5 for GBS-negative (12/KP, 13/KP, 24/KP, 28/KP, 29/KP), as a negative control, to confirm their high immunoreactivity to every tested serum and to avoid false results, which could occur in the pooled sera.

### Statistical analysis

Statistical analyses were carried out by Student's *t*-test for independent samples, Dunnett's multiple comparisons test and one-way analysis of variance (ANOVA) using IBM SPSS Statistics 24. *P* values <0.05 were considered statistically significant.

## Results

In our previous study at least four immunoreactive proteins, including enolase, were identified in a pool of sixty genetically different GBS strains isolated from various infection types (Brzychczy-Wloch et al., 2013). In this paper, we extended the number of bacterial samples tested (*n* = 180) and included umbilical cord serum (*n* = 34).

### Immunoreactive proteins identification and epitopes mapping

**Figure 1:**
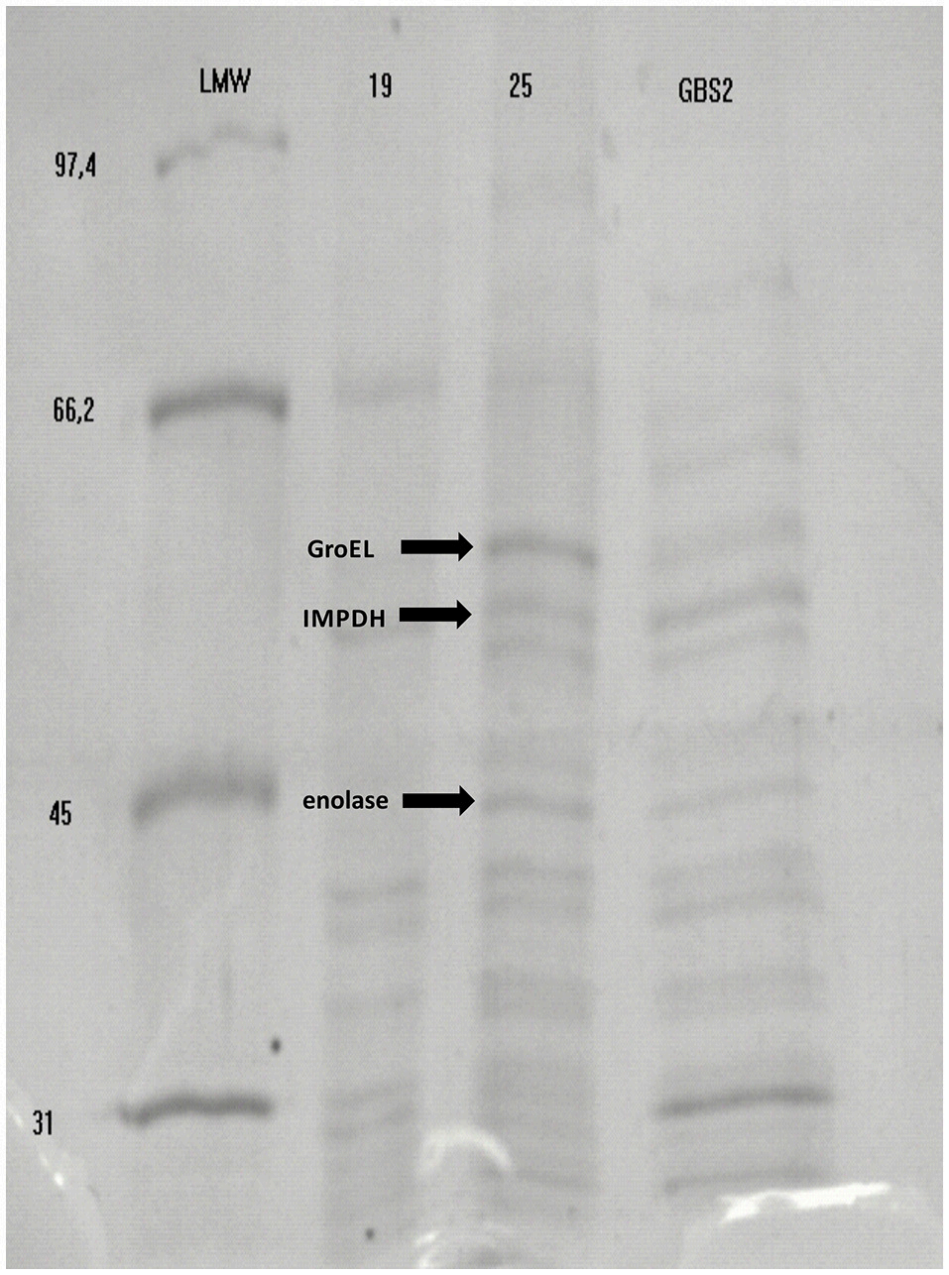
Example results of detection of immunoreactive *S. agalactiae* proteins: enolase protein (45 kDa), inosine 5′-monophoshate dehydrogenase (53 kDa) and molecular chaperone GroEL (57 kDa) separated in SDS-PAGE and identified by Mascot (Matrix Science, London, United Kingdom). Legend: Lane 1, marker LMW (low molecular weight) (GE Healthcare Life Sciences); Lane 2, GBS strain 19-2337/08 isolate from newborn's mouth smear; Line 3, GBS isolate 25–CM/47 isolate from neonatal venous blood; Lane 4, GBS isolate GBS 2 isolated from vagina.

Using Heilmann's modified method, followed by immunoblotting we isolated and detected at least six immunoreactive proteins in all GBS strains with molecular masses ranging between 45 and 57 kDa. Nevertheless, three of which are studied in this paper: enolase (47.4 kDa), IMPDH (53 kDa), and molecular chaperone GroEL (57 kDa), in all GBS strains (Figure 1).

Prediction of the most probable epitopes, based on bioinformatic analyses, allowed us to choose peptides consisting of 6–16 amino acids as follows: for enolase protein: 32 peptides, for IMPDH: 36, and for GroEL: 41. Chemically, epitopes are defined as a part of the protein consisting of dozens of amino acids, which is a peptide. The peptides were selected for further investigation involving the PEPSCAN synthesis (Table 1).

**Table 1:** List of the most likely epitopes of the *S. agalactiae* immunoreactive proteins: enolase, inosine 5′-monophosphate dehydrogenase (IMPDH) and molecular chaperone GroEL.

**No**.	**Enolase**	**No**.	**Inosine 5′-monophosphate dehydrogenase**	**No**.	**Molecular chaperone GroEL**
1	^4^VYAREVLDSRGNPT^17^	1	^1^MSNWDTKFLKKGFT^14^	1	^16^VRGVDILADTVKV^28^
2	^17^TLEVEVYTESGAFG^30^	2	^15^FDDVLLIPAESHVLP^29^	2	^29^TLGPKGRNVVLEKA^42^
3	^34^VSGASTGEHEAVELR^48^	3	^30^NEVDMNTKLA^39^	3	^41^KAFGSPLITN^50^
4	^48^RDGDKSRYGGLGTQ^61^	4	^44^LNIPIITAAMDTVTD^58^	4	^53^VTIAKEIELEDHF^65^
5	^61^QKAVDNVNNVIAEA^74^	5	^69^GGLGIIHKNMSIVD^82^	5	^70^AKLVSEVASKTN^81^
6	^75^IIGYDVRDQQAI^86^	6	^83^QAEEVRKVKRSEN^95^	6	^82^DIAGDGTTTAT^92^
7	^88^RAMIALDGTPNKG^101^	7	^96^GVIIDPFFLT^105^	7	^91^ATVLTQAIVRE^101^
8	^102^KLGANAILGVSIAVA^116^	8	^104^LTPDNTVSEAEELMQ^118^	8	^105^NVTAGANPIGI^115^
9	^117^RAAADYLEVPLYSYLG^131^	9	^119^NYRISGVPIVET^130^	9	^116^RRGIETAVSAAVEEL^130^
10	^135^TKVLPT^140^	10	^130^TLENRK^135^	10	^132^EIAQPVSGKEA^142^
11	^148^GSHSDAPIAFQEF^161^	11	^148^ISDYKQLISE^157^	11	^143^IAQVAAVSSRSEKVGE^158^
12	^162^MIMPVGAPTFKEAL^175^	12	^162^QNLVTAPIGTDLE^168^	12	^165^ERVGND^170^
13	^178^AEVFHALKKI^188^	13	^178^RILHEH^183^	13	^172^VITIEESRGME^182^
14	^194^LETAVGDEGGFAPK^207^	14	^185^IEKLPLVDDEGR^196^	14	^183^TELEVVEGMQFDRGY^197^
15	^208^FEGTEDGVETILKAI ^222^	15	^198^SGLITIK^204^	15	^198^LSQYMVTDNEKMV^210^
16	^224^AAGYEAGE^231^	16	^206^IEKVIEFPKAAKDEF^213^	16	^211^SELENPYILITD^222^
17	^237^GFDCASSEFYD^245^	17	^221^GRLLVAGAVG^229^	17	^228^IQEILPLLEEV^238^
18	^243^SEFYDAERKVYDYS^256^	18	^230^GVTSDTFERA^239^	18	^238^VLKTNRPLLIIADD^251^
19	^257^KFEGEGGAVRTAAE^270^	19	^239^AEALFEA^245^	19	^251^DVDGEALPTLVLNK^264^
20	^271^QIDYLEELVNKYP^283^	20	^248^DAIVIDTA^255^	20	^267^GTFNVVAVKAPG^278^
21	^284^IITIEDGMDENDWD^297^	21	^256^HGHSAGVLRKI^266^	21	^276^APGFGDRRKAM^286^
22	^298^GWKALT^305^	22	^284^ATAEGARALYDAGVD^298^	22	^288^EDIAILTGGTVVTED^302^
23	^308^GRVQLVGDDF^317^	23	^299^VVKVGIGPGSICTTR^313^	23	^312^MQVLGQSAKVTVDKD^326^
24	^328^GIKEEAA^334^	24	^314^VVAGVGVPQITAIYD^328^	24	^326^DSTVIVEGAGDSSAI^340^
25	^334^ANSILIKVNQI^344^	25	^329^AAAVAREYGKTII^341^	25	^341^ANRVAIIK^348^
26	^359^EAGYTAVVSHR^369^	26	^345^GIKYSGDIVKALAA^357^	26	^349^SQMEATTSDFDRE^361^
27	^368^HRSGETEDSTI^378^	27	^370^AGTDEAPGETEIF^382^	27	^362^KLQERLAKLA^371^
28	^378^IADIAVATN^386^	28	^382^QGRKFKTYRG^392^	28	^371^AGGVAVIKVGAA^382^
29	^386^NAGQIKTGSLSR^397^	29	^399^MKKGSSDRYFQGSVN^413^	29	^381^AATETELKEMKLR^393^
30	^393^GSLSRTDRIAKYNQ^406^	30	^414^EANKLVPEGIE^424^	30	^403^AAVEEGIVSGGGT^415^
31	^404^YNQLLRIE^411^	31	^425^GRVAYKGSVAD^435^	31	^415^TALVNVIEKVAALKL^429^
32	^414^LGEVAQYKGIK^424^	32	^436^VFQMLGGIR^445^	32	^430^NGDEETG^436^
		33	^446^SGMGYVGAAN^455^	33	^438^NIVLRALEEPVRQIA^452^
		34	^457^KELHDNAQFVEM^468^	34	^452^AYNAGYEGSVIIERL^466^
		35	^473^LKESHPHDVQIT^484^	35	^464^ERLKQSE^470^
		36	^485^NEAPNYSVH^493^	36	^469^SEIGTGFNAANGEWV^483^
				37	^485^MVTTGIIDPVKV^496^
				38	^495^KVTRSALQNA^504^
				39	^501^LQNAASVASLILTTE^515^
				40	^516^AVVANKPEPEAP^527^
				41	^427^PTAPAMDPSMM^537^

### Immunoreactivity of the epitopes

**Figure 2:**
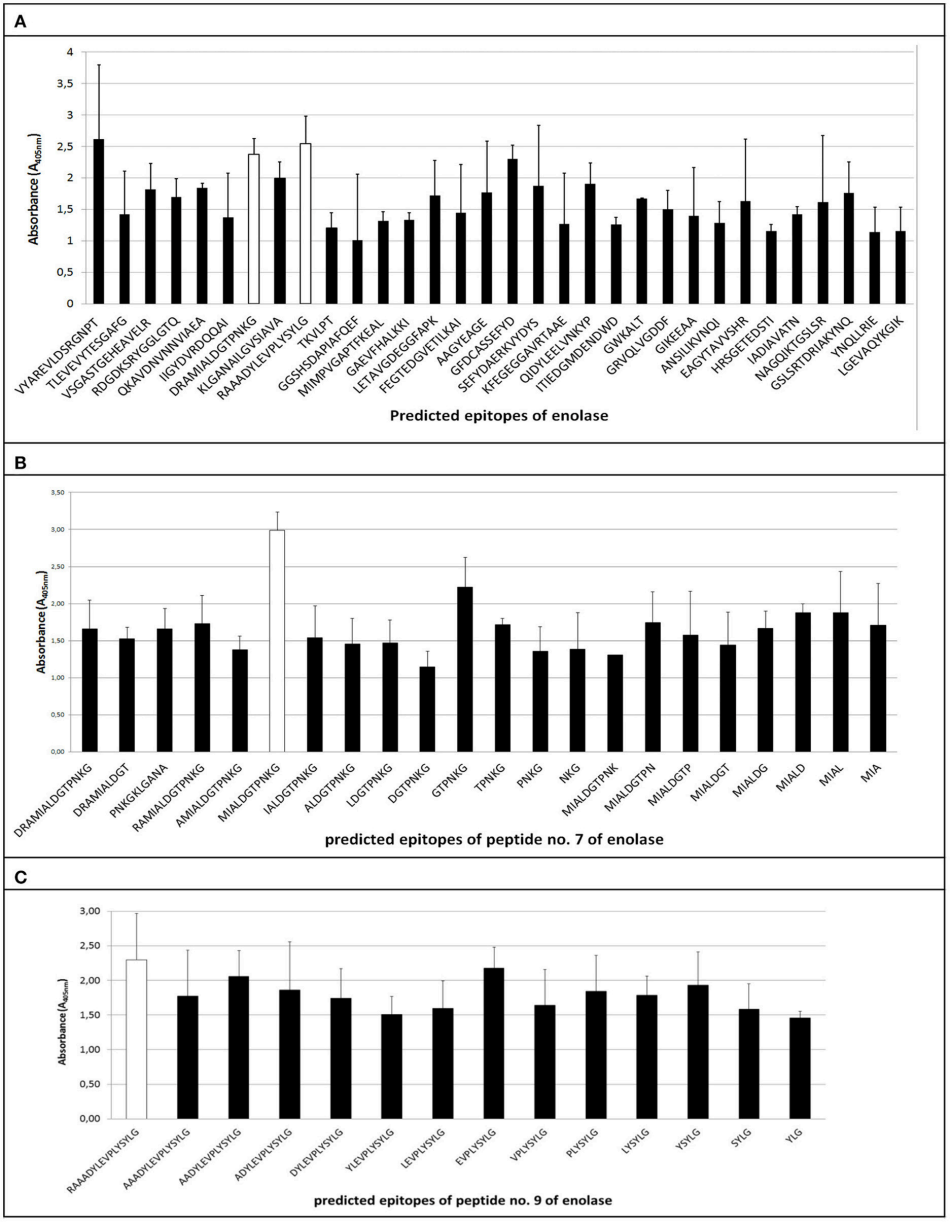
Mapping of epitopes among enolase protein synthesized on polyethylene pins by PEPSCAN. Two peptides (peptides no. 7 and no. 9). **(A)** showed the highest absorbance to pooled GBS-positive umbilical cord blood sera and low standard deviation. **(B,C)** show mean absorbance values of peptides received in modified synthesis based on cutting off consecutive amino acids from C-end and/or N-end.

**Figure 3:**
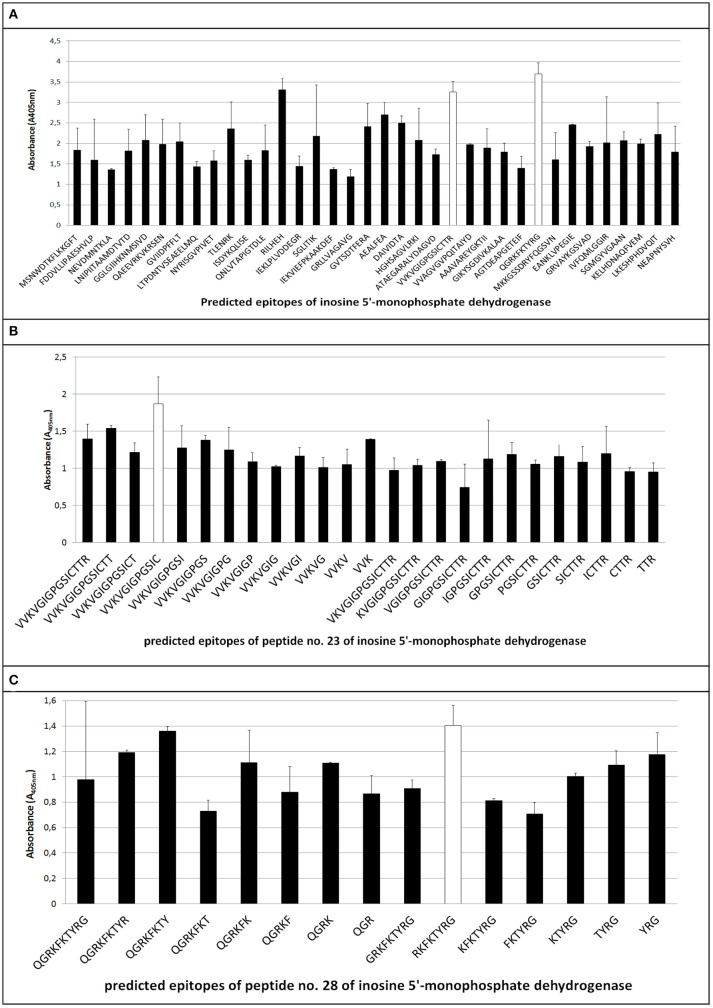
Mapping of epitopes among inosine 5′-monophosphate dehydrogenase synthesized on polyethylene pins by PEPSCAN. Two peptides (peptides no. 23 and no. 28) **(A)** showed the highest absorbance in the presence of pooled GBS-positive umbilical cord blood sera, thus were chosen for further analyses based on cutting off consecutive amino acids from C- and N- end **(B,C)**.

**Figure 4:**
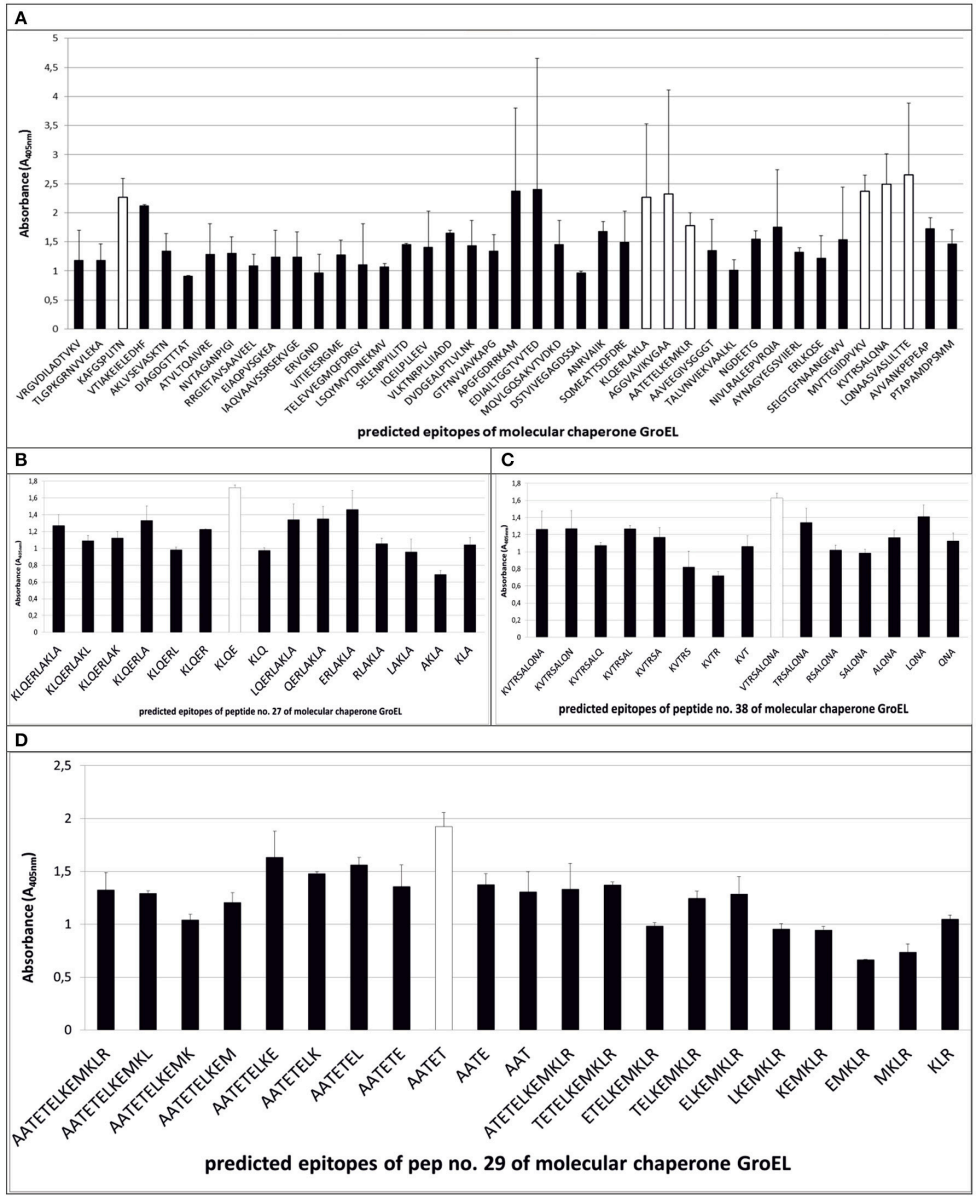
Mapping of epitopes among molecular chaperone GroEL synthesized on polyethylene pins by PEPSCAN. Seven peptides (peptides no. 3, no. 27, no. 28, no. 29, no. 37, no. 38, and no. 39) **(A)** showed the highest absorbance in the presence of pooled GBS-positive umbilical cord blood sera. **(B–D)** show detection of the shortest peptides highly immunoreactive to GBS-positive sera on the example of peptides no. 27, no. 29, and no. 38.

The ELISA assay performed after synthesis allowed us to identify 11 peptides with the highest immunoreactivity measured by absorbance value: two in enolase - ^88^DRAMIALDGTPNKG^101^ (peptide no. 7), ^117^RAAADYLEVPLYSYLG^131^ (peptide no. 9) (Figure 2A); two in IMPDH - ^299^VVKVGIGPGSICTTR^313^ (peptide no. 23), ^382^QGRKFKTYRG^392^ (peptide no. 28) (Figure 3A); and seven in molecular chaperone GroEL—^41^KAFGSPLITN^50^ (peptide no. 3), ^362^KLQERLAKLA^371^ (peptide no. 27), ^371^AGGVAVIKVGAA^382^ (peptide no. 28), ^381^AATETELKEMKLR^393^ (peptide no. 29), ^485^MVTTGIIDPVKV^496^ (peptide no. 37), ^495^KVTRSALQNA^504^ (peptide no. 38), ^501^LQNAASVASLILTTE^515^ (peptide no. 39) (Figure 4A). Most of them, except ^88^DRAMIALDGTPNKG^101^ (enolase, peptide no. 7), ^371^AGGVAVIKVGAA^382^ (GroEL, peptide no. 28), ^362^KLQERLAKLA^371^ (GroEL, peptide no. 27), and ^501^LQNAASVASLILTTE^515^ (GroEL, peptide no. 39) demonstrated significant specificity (Table 2). Although the ^4^VYAREVLDSRGNPT^17^ (enolase, peptide no. 1) sequence presented the highest absorbance, it was dismissed in further analysis due to the highest standard deviation.

**Table 2:** List of the most immunoreactive peptides of enolase, inosine 5′-monophosphate dehydrogenase and molecular chaperone GroEL (A) and epitopes received after modification based on cutting-off consecutive amino acids to obtain the shortest immunoreactive sequence (B) and their mean absorbance value measured in presence of pooled GBS-positive (GBS+) sera and pooled GBS-negative (GBS−) sera.

**Protein**	**Peptide**	**A405 nm (GBS+) [M ±SD]**	**A405 nm (GBS−) [M ±SD]**	***p* value**
**A**				
Enolase	RAMIALDGTPNKG	2.68 ± 0.18	0.06 ±0.05	0.027
	RAAADYLEVPLYSYLG	1.81 ± 0.41	0.05 ±0.05	0.103
Inosine 5′-monophosphate dehydrogenase (IMPDH)	VVKVGIGPGSICTTR	3.26 ± 0.25	0.92 ±0.20	0.009
	QGRKFKTYRG	3.70 ± 0.27	0.47 ±0.61	0.021
Molecular chaperone GroEL	KAFGSPLITN	2.27 ± 0.33	1.01 ± 0.11	0.036
	KLQERLAKLA	2.27 ± 1.26	0.94 ± 0.13	0.275
	AGGVAVIKVGAA	2.32 ± 1.79	0.69 ± 0.00	0.327
	AATETELKEMKLR	1.77 ± 0.23	0.68 ± 0.16	0.031
	MVTTGIIDPVKV	2.37 ± 0.28	0.92 ± 0.01	0.018
	KVTRSALQNA	2.49 ± 0.52	0.63 ± 0.21	0.043
	LQNAASVASLILTTE	2.65 ± 1.23	1.01 ± 0.04	0.201
**B**				
Enolase	MIALDGTPNKG	2.05 ± 0.50	0.54 ± 0.49	0.002
Inosine 5′-monophosphate dehydrogenase (IMPDH)	VVKVGIGPGSIC	1.87 ± 0.30	0.54 ± 0.36	0.001
	RKFKTYRG	1.40 ± 0.04	2.42 ± 0.16	0.004
Molecular chaperone GroEL	SVASLILTTE	1.50 ± 0.01	0.32 ± 0.10	0.001
	FGSPLITN	1.69 ± 0.12	0.83 ± 0.50	0.110
	MVTTGIIDPVK	1.93 ±0.30	0.65 ± 0.54	0.004
	AGGVA	1.82 ± 0.11	0.78 ± 0.21	0.024
	KLQE	1.72 ± 0.05	0.66 ± 0.03	0.002
	AATET	1.92 ± 0.28	0.31 ± 0.13	<0.001
	VTRSALQNA	1.63 ± 0.26	0.33 ± 0.06	0.080

*Statistical significance in immunoreactivity to both GBS-positive and GBS-negative sera described by Student's t-test*.

### Epitopes modification on their immunoreactivity

**Figure 5:**
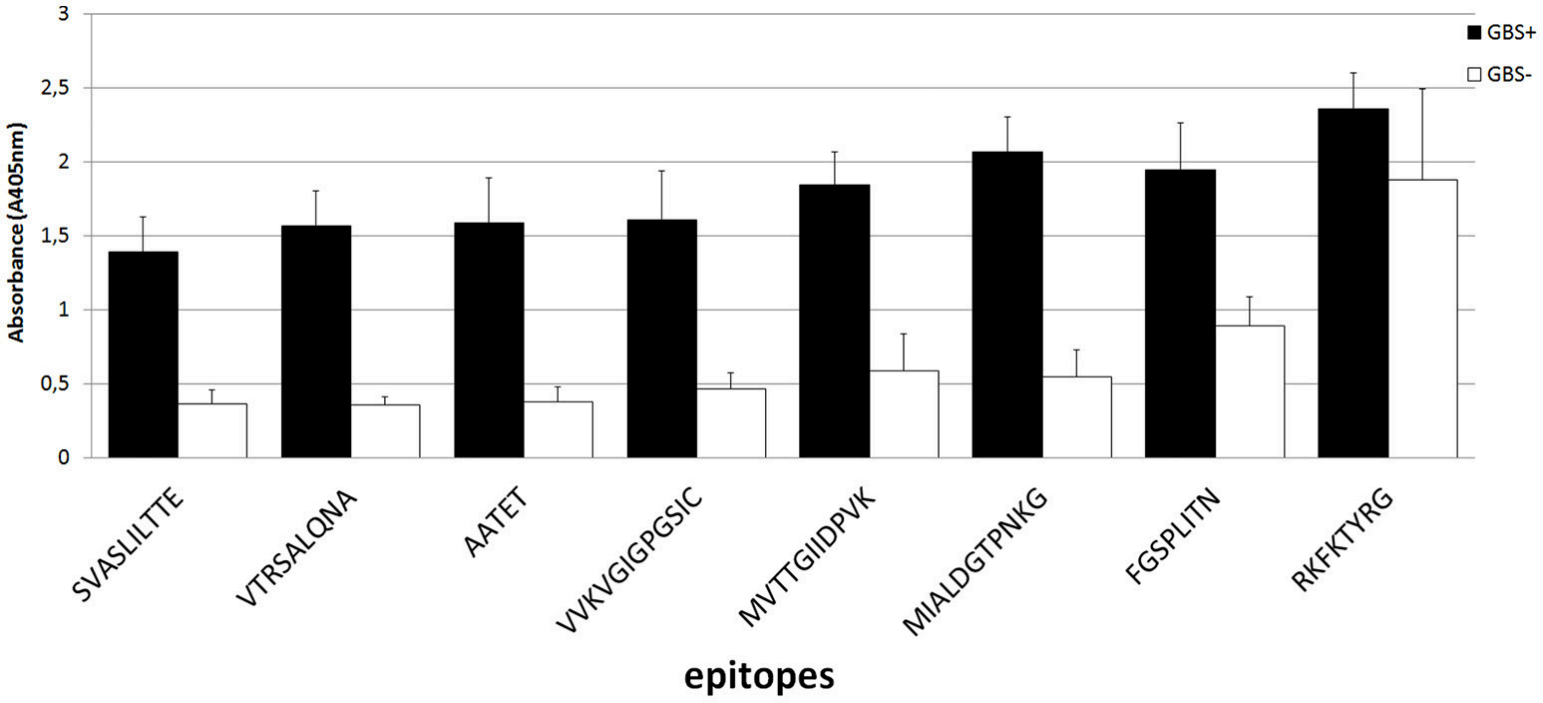
List of the derivatives of epitopes received in modified synthesis based on cutting off consecutive amino acids. Mean absorbance values were measured in the presence of single GBS-positive serum (*n* = 10) and GBS-negative serum (*n* = 4). Specificity of the epitopes identified was determined by Student's *t*-test.

The epitopes listed were modified by cutting off consecutive amino acids from N-end and C-end to obtain the shortest immunogenic sequence specifically reactive to the serum (Figure 5). To sum up, for enolase, among the 36 peptides tested, two ^91^MIALDGTPNKG^101^ and ^117^RAAADYLEVPLYSYLG^131^ demonstrated the highest immunoreactivity to pooled GBS-positive serum and weak reactivity with pooled GBS-negative serum (Figures 2B,C, 5). In IMPDH, 40 peptides were tested, among which two: ^299^VVKVGIGPGSIC^310^ and ^385^RKFKTYRG^392^ demonstrated the highest immunoreactivity to the tested serum (Figure 3C,B, 5). In GroEL, 194 peptides were tested, among which seven: ^43^FGSPLITN^50^, ^362^KLQE^365^, ^371^AGGVA^375^, ^381^AATET^385^, ^485^MVTTGIIDPVK^495^, ^496^VTRSALQNA^504^, and ^505^SVASLILTTE^515^ were qualified as potential epitopes (Figures 4B,C,D, 5). Removing consecutive amino acids of ^117^RAAADYLEVPLYSYLG^131^ did not result in an increase in immunoreactivity in any derivative peptide, therefore, it was qualified as the epitope (Figure 2C). The peptides listed were subsequently examined with single sera, both GBS-positive and GBS-negative, to determine their selectivity. All of the epitopes tested, except ^385^RKFKTYRG^392^, demonstrated significantly high immunoreactivity to all GBS-positive sera in comparison to GBS-negative sera (Figure 5), which confirmed their selectivity and allowed to classify them as epitopes.

Another modification of the peptide synthesis carried out in this paper was substitution of consecutive amino acid by alanine and/or glycine. The aim of this modification was to determine the impact of each amino acid in the induction of immunoreactivity. Among all (*n* = 10) of the tested peptides, only in one epitope of GroEL protein, ^485^MVTTGIIDPVKV^496^, substitution with alanine at position 486 resulted in significant growth of immunoreactivity to GBS-positive serum. Nevertheless, every substitution with alanine, except isoleucine at position 490, resulted in higher absorbance in comparison to the initial epitope.

## Discussion

Despite the popularity of intrapartum antibiotic prophylaxis, infections caused by GBS still result in high morbidity and mortality among newborns. Additionally, the overuse of antibiotics causes the increase of bacterial resistance to a growing number of antibiotics (Libster et al., 2012). Quick diagnostic of GBS infections could maximally shorten the waiting time for results, which would accelerate the initiation of targeted antibiotic therapy or, in the case of a negative result, avoid unnecessary administration of antibiotics. Currently, there is no quick, effective and precise diagnostic immunoassay for *S. agalactiae* infections and/or carriage. To develop a precise and quick diagnostic test for pathogenic GBS, an immunoreactive protein specific to GBS could be used. In our paper, we identified three such proteins which were enolase, IMPDH and molecular chaperone GroEL.

Enolase is a glycolytic enzyme which plays an important role in bacterial pathogenesis. This anchorless surface protein, which binds plasminogen, is common for the most of streptococcal groups (Pancholi and Fischetti, 1998). Even though immunoreactive nature of GBS enolase has been previously described, there is still lack of detailed knowledge of its epitopes (Hughes et al., 2002; Fluegge et al., 2004).

As a result of protein identification followed by the bioinformatic analysis of enolase, 32 peptides (Table 1) defined as potential epitopes were subjected to the PEPSCAN method synthesis (Geysen et al., 1987). The length of the chosen peptides ranged from 8 to 16 amino acids, and the selection of individual peptides was determined according to the prediction methods described above. Introducing this modification allowed to single out two potential epitopes: peptide no. 7 and peptide no. 9 (Figure 2A), which showed the highest absorbance in reaction with GBS-positive sera, a very low reactivity level with GBS-negative sera and a low standard deviation (Table 2). As a result of modified synthesis, based on cutting off consecutive amino acids to determine the shortest immunogenic sequence, derivative of the peptide no. 9, which was ^91^MIALDGTPNKG^101^ demonstrated the highest immunoreactivity to GBS-positive serum (selectivity *p* = 0.02) (Figure 5), modification of peptide no. 7 did not show any growth in immunoreactivity (Figure 2C). Even though the enolase peptides selected are common for numerous bacterial species (https://www.ncbi.nlm.nih.gov/BLAST/), without any doubt, they are specifically recognized by GBS-positive sera (*p* = 0.02).

LaFrentz et al. have used enolase as a vaccine component given to *Nile tilapia* to protect it from infection caused by *Streptococcus iniae* (LaFrentz et al., 2011). Research on epitopes of *Clonorchis sinensis* enolase isolated by Wang et al. allowed to identify 10 epitopes and proved their immunogenicity (Wang et al., 2014). Dutta et al., working on *Plasmodium falciparum*, identified one epitope, ^104^EWGWS^108^, in enolase and validated its protective role in mice model (Dutta et al., 2015). In turn, Kolberg et al. identified another epitope, ^55^DKSRYGGLG^63^, in *S. pneumoniae* enolase (Kolberg et al., 2006). Nevertheless, the current state of our knowledge indicates that no one has thus far identified the same epitopes as the ones described in our work. The differences among the presented trials may be a confirmation of uniqueness of the epitopes identified.

The second protein analyzed was inosine 5′-monophosphate dehydrogenase (IMPDH). This protein is stable tetrameric purine, which is known for the catalysis of the key stage of *de novo* guanine nucleotide synthesis in all organisms, consequently an essential precursor for DNA and RNA synthesis (Cohen and Sadee, 1983; Hedstrom, 2009). IMPDH, playing a crucial role in cell replication is targeted in antiviral, antibacterial or anticancer therapies, as well as in auto immunological disease treatment (Ratcliffe, 2006; Hedstrom, 2009; Juda et al., 2014; Shah and Kharkar, 2015).

The use of the appropriate prediction method preceded by the bioinformatic analysis of the IMPDH allowed us to identify 36 peptides (Table 1), the sequence length of which oscillated between 6 and 15 amino acids and presented features of potential epitopes, whereas 2 of them (Table 2) showed the highest immunoreactivity. Two peptides: peptide no. 23 and peptide no. 28 were eventually singled out and subjected to further analyses (Figure 5). Peptide no. 28 presented high immunoreactivity both to GBS-positive and GBS-negative sera, therefore it was dismissed from further investigation (Figure 5). The analysis of the sequences of both peptides (no. 23 and no. 28) in NCBI BLAST database showed that they are representative of only one protein, which is IMPDH. Thus far, IMPDH has been examined for its immunogenicity in other species. Pitarch et al. showed that IMPDH is one of the immunogenic proteins in *Candida albicans* (Pitarch et al., 2001), whereas Duan et al. suggested its immunogenicity in *Streptococcus suis* (Duan et al., 2006). Therefore, it proves that our discovery of the immunogenic role of this conservative GBS protein is, to our knowledge, pioneering.

The third protein subjected to our investigation was one that is common in most bacteria, the molecular chaperone GroEL, which belongs to the chaperonin family. GroEL is a double ring tetradecamer, consisting of seven subunits in *cis* and *trans* positions, which show ability to form barrel-like structures with hydrophilic cavities (Zeilstra-Ryalls et al., 1991; Mayhew et al., 1996). The key role of this protein is its contribution to the folding of other proteins, therefore it shows both functional and structural similarity to human Hsp60 (Horwich et al., 2007). As an equivalent of heat shock protein, GroEL also shows its immunogenic character (Kaufmann, 1990; Shinnick, 1991), however similarly to IMPDH, its immunogenic role in *S. agalactiae* has not been described yet.

In our study, 41 peptides of the GroEL (Table 1) have been subjected to synthesis, and according to the bioinformatic analysis conducted, have been singled out as potential epitopes. The ELISA assay allowed to determine seven polypeptides that indicated the highest immunoreactivity (Table 2). Peptide no. 29 actually did not show the highest absorbance, nevertheless it was subjected to further analysis, because its sequence coincided with peptide no. 28 with two amino acids at positions 381–382. The aim was to determine whether the epitope coincided with both peptides. A modified synthesis based on cutting off the consecutive amino acids allowed to identify seven potential epitopes (Figure 5). Even though, according to the literature, peptides with fewer than five amino acids should not be considered epitopes, we did not dismiss epitope ^262^KLQE^265^ from further analyses, because it was characterized by high immunoreactivity and selectivity (Benjamini et al., 1997). Interestingly, derivatives of peptides no. 27 and no. 28 presented significant specificity to the tested sera. To our best knowledge, there has not been such detailed research on the epitopes of the GroEL isolated from GBS thus far. However, results of research conducted on the whole protein isolated from species such as *Salmonella typhi, Francisella tularensis*, or *Porphyromonas gingivalis* were previously described (Panchanathan et al., 1998; Maeda et al., 2000; Valentino et al., 2011). This proves the immunogenic character of this protein and, at the same time, the differences between the epitopes identified among this protein can indicate its species-specificity in the context of the future rapid diagnostic test for GBS infections. The analysis of the chosen epitopes in the NCBI Protein BLAST base showed that all peptides, except the peptide no. 28 sequence, which was also identified in the protein adenylosuccinate lyase, were characteristic exclusively of the GroEL.

We would like to underline that our study concerns on epitopes not on whole protein. The epitopes (chemically short peptides) were selected experimentally. They react selectively only with GBS-positive sera. However, we have made experiments with additional sera of a carrier *Streptococcus pyogenes* (GAS). The results clearly showed no reactivity with the epitopes comparing to the reactivity with GBS-positive sera. We would like to emphasize, that the presented results are a quite new scientific report about the possibility of epitope use as a very useful tool to develop a new-quality diagnostic test to detect *S. agalactiae* bacteria. However, it must be emphasized that in the test compilation of different epitopes (from different immunoreactive) proteins will be used and the test condition needs to be optimized. Received results constitute the basis for further investigation on highly specific immunoassay.

There is still no universal, rapid, precise diagnostic immunoassay for identification of infection caused by GBS as well as recognizing carriage of these bacteria. We believe that an immunoassay such as ELISA or Western Blot, based on immunoreactive proteins, or preferably, their highly specific epitopes, would reduce waiting time for results, which would allow for immediate application of antibiotic therapy. Therefore, in our study, we attempted to identify immunoreactive proteins of GBS and their epitopes selectively reacting with antibodies from umbilical cord blood. We have identified three immunogenic proteins and have shown the immunogenic nature of IMPDH and molecular chaperone GroEL in GBS for the first time. Additionally, we have selected and synthesized ten epitopes from the 266 amino acid sequences tested, which demonstrated selectivity and the highest immunoreactivity to umbilical cord antibodies. Those features can qualify them as potential markers for GBS infections as well as components of the innovative subunit vaccine dedicated to pregnant women, the elderly and chronic disease patients, which would contribute to the reduction of the effects caused by *S. agalactiae*. However, there is no doubt that extensive advanced research must also be undertaken.

## Author contributions

AD conducted peptide synthesis, carried out ELISA assays, contributed reagents or materilas and drafted the manuscript, EB coordinated peptide synthesis and helped to draft the manuscript, SG designed, coordinated, and conceived the study, performed the protein identification and helped to draft the manuscript, MP carried out protein isolation and statistical analysis, EM performed the study population and specimen collection, AG was a supervisor and helped to draft the manuscript, MB provided an apparatus and laboratories, MB-W performed the identification of bacterial strains, designed, coordinated, and helped to draft the manuscript. All authors read and approved the final manuscript.

### Conflict of interest statement

The authors declare that the research was conducted in the absence of any commercial or financial relationships that could be construed as a potential conflict of interest.
